# THE BALTIMORE LONGITUDINAL STUDY OF AGING: READY TO SHARE

**DOI:** 10.1093/geroni/igac059.1230

**Published:** 2022-12-20

**Authors:** Eleanor Simonsick, Luigi Ferrucci

## Abstract

Beginning January 2023, the new National Institutes of Health (NIH) Data Sharing Policy for NIH-funded and conducted research is scheduled to go into effect. In anticipation of this requirement, scientists and staff of the Baltimore Longitudinal Study of Aging (BLSA) launched a comprehensive effort to create a user friendly well-documented accessible database scheduled for a “soft” release just in time for GSA. The BLSA, established in 1958 to study normative aging, continues to advance knowledge and understanding of aging mechanisms and their impact on and response to disease processes. During a 3-day clinic visit, the 1200+ BLSA participants undergo comprehensive evaluation covering the full range of physical functional and cognitive performance capacity as well as extensive imaging studies, biological sample collection and clinical examination. In this symposium, Dr. Griswold will introduce the data sharing platform and demonstrate its facility. Dr. Simonsick will elaborate on development of a fitness percentile-based approach to define exceptional aging and the factors associated with maintaining exceptional status. Dr. Moore will provide findings on life-course physical activity and CT derived muscle parameters and homeostatic regulation in later life. Dr. Tian will share her ongoing work aimed at identifying metabolomic signatures of brain atrophy and Alzheimer’s disease risk. Dr. Tanaka will report on her work analyzing the metabolomic profile of different dietary patterns and their association with frailty. Our Discussant, Dr. Ferrucci will briefly share future directions for the BLSA and then invite attendees to comment on the BLSA data sharing platform and scientific priorities.

